# Effect of Public Fillers on Cement-Stabilized Recycled Mixes of Road Performance: Mechanical Properties, Microstructure, and Durability

**DOI:** 10.3390/ma17092018

**Published:** 2024-04-26

**Authors:** Ming Zhang, Chen Cheng, Kingsley Chiang, Xinxin Wang, Yazhi Zhu, Hui Luo

**Affiliations:** 1 China State Construction Engineering (Hong Kong) Limited, Hong Kong 999077, China; zhangming@cohl.com (M.Z.); chen_cheng@cohl.com (C.C.); kingsleychiang@cohl.com (K.C.); zjwangxinxin@cohl.com (X.W.); 2 Department of Structural Engineering, Tongji University, Shanghai 200092, China; 3 School of Civil Engineering, Jiangsu Ocean University, Lianyungang 222005, China; water@njfu.edu.cn

**Keywords:** public fill, mechanical properties, replacement ratio, shrinkage property, stabilization, microstructure

## Abstract

In order to address the challenges of resource utilization posed by construction waste, the substitution of natural aggregate (NA) with public fill (PF) contents was investigated for load reclamation and road grassroots applications. A comprehensive assessment of road performance for the recycled mixture was conducted, focusing on parameters such as unconfined compressive strength, splitting strength, compressive resilience modulus, dry shrinkage, and frost resistance. Additionally, the impact of incorporating PF at various types and replacement ratios on the microstructure of cement-stabilized aggregate (CSA) was analyzed. The results indicated that the unconfined compressive strength of cement-stabilized recycled mixture with varying PF contents meets the base strength requirements for heavy, medium, and light traffic pavement on secondary and sub-secondary roads in China. Notably, the unconfined compressive strength and resilience modulus follow a similar pattern, reaching their peak at a 25% PF content. Microscopic examination reveals that an appropriate PF content leads to the predominant formation of C(N)-A-S-H, hydrotalcite, Ca(OH)_2_, and CaCO_3_ as paste reaction products. As the replacement of public fill increases from 0% to 25%, there is a gradual stacking of gel products, which enhances the compactness of the microstructure by cementing together unreacted particles. Consequently, this process reduces dry shrinkage strain and effectively mitigates the formation of reflection cracks. Applying large quantities of public fill to road construction can effectively deal with various waste accumulation problems and produce a novel road material with significant social, economic, and environmental benefits.

## 1. Introduction

The escalating pace of urbanization has led to a steady rise in the cumulative volume of construction waste emanating from urban infrastructure expansion, road reconstruction, and the dismantling of structures. This upward trend presents a formidable obstacle in efforts to eradicate the prevalent practice of landfill disposal for construction waste, thus exacerbating environmental concerns [1,2]. Over ninety percent of construction waste consists of solidified slag, red brick, mortar, and concrete waste, commonly referred to as public fill (PF) because of its inert nature [3]. It is important to note that PF is not hazardous and can be reused in construction projects. Crushing these public fills into recycled aggregate as a substitute for natural aggregate (NA) is an effective way to mitigate the amount of construction waste. Various experiments have been conducted by researchers on the macroscopic properties, microscopic aggregation morphology, and microscopic mineral composition of PF, with results indicating that PF is characterized by high porosity, small apparent density, unstable material source, and high variability, which is easy to break [4,5,6]. The properties of PF are not as good as those of NA, and the mixture of PF can negatively affect the rheological properties, mechanical properties, and durability of concrete. Various curing agents, including liquid alternatives, were employed by researchers to augment the properties of cement-stabilized materials. Their investigation revealed that the unmodified cement-stabilized material satisfied the specifications for grade II and lower road bases. Conversely, the modified formulations demonstrated enhanced strength properties, meeting the standards for grade I and primary highway bases. Recyclable public fill has been widely applied for preparing recycled concrete and sustainable alkali-activated materials, and previous studies have reported some satisfactory results [7]. In particular, alkali-activated concrete incorporated with recycled aggregates has environmental benefits and good performance to meet the requirements of low-carbon and green building materials, significantly promoting the engineering application of recycled concrete [8,9]. Furthermore, the distinctions between PF and NA regarding edge angle, sphericity, and mineral composition provide a solid basis for utilizing PF in road construction.

By 2023, the aggregate length of highways across China extended to 5.35 million kilometers. This expansion has escalated the requirement for non-renewable resources, notably sand and gravel, thereby amplifying both highway construction and maintenance expenditures. Consequently, the upward trend in costs has impeded the progress of the highway transportation sector [10]. Cement-stabilized aggregate (CSA) is a commonly used semi-rigid base material in the design and construction of highway pavements [11]. Both CSA and concrete are cementitious materials. However, reflection cracking is a common issue in asphalt pavements due to the poor cracking resistance of CSA. De Maio et al. [12] proposed a numerical model to investigate the structural response of nano-enhanced fiber-reinforced polymer (FRP)-plated reinforced concrete (RC) beams in terms of load-carrying capacity, crack pattern evolution, and failure mode. This model was also proved to be applicable for damage detection of failure modes such as concrete crushing and rebar yielding of other structures [13,14]. To improve the crack resistance of CSA, adding a suitable amount of public fill particles such as construction waste can reduce the use of natural sand and gravel while enhancing its structural integrity. Therefore, public fill emerges from the crushing, screening, and processing of construction waste, facilitating its utilization in the construction of low-level roads [15,16,17]. This methodology yields substantial ecological, economic, and societal advantages, thereby playing a pivotal role in advancing China’s ‘dual-carbon strategy’ and fostering rural revitalization endeavors.

Prior research has highlighted the detrimental impact of construction waste on the strength characteristics of CSA [18,19]. Zhang et al. [20] conducted experiments involving the segregation of recycled concrete aggregate (RCA) and crushed bricks (RCB) from recycled aggregate (RA) to formulate cement-stabilized recycled mixtures. Their findings indicated suitability for primary road bases, provided the RA content remained below 50%, with RCB/RCA proportions not exceeding 3:7. Similarly, Disfani et al. [21] conducted laboratory investigations on recycled cement-stabilized mixtures, revealing that the physical, mechanical, and fatigue resistance properties of the recycled mixtures complied with Highway Authority standards when employing 5% cement and less than 50% RCB. Zhao et al. [22] replaced different percentages of aggregates with waste particles (1.18~2.36 mm and 2.36~4.75 mm) and observed a decrease in the indirect tensile strength of CSA with increasing waste content and rubber particle size. Farhan et al. [23] investigated the impact of volume substitution rates of three types of waste (15%, 30%, and 45%) on mechanical properties. They observed a decrease in indirect tensile strength by 8.08%, 23.67%, and 24.24%, respectively. Similarly, the flexural tensile strength and unconfined compressive strength exhibited adverse effects with increasing waste particle content, surpassing the influence of waste particle size. Prior research indicates that the decline in mechanical properties of recycled cement-stabilized aggregate (RCSA) can be attributed, in part, to water absorption by recycled aggregate and the presence of bonding defects at the cement matrix interface [24,25]. Therefore, researchers have explored different pretreatment methods such as sodium hydroxide, styrene-butadiene latex, sodium sulfate solution, and silane coupling agent for rubber. Their findings revealed enhanced bonding between treated recycled aggregate and the cement matrix, resulting in improved mechanical properties and freeze–thaw resistance. Despite the reduction in RCSA strength, the toughness, energy absorption capacity, and fatigue resistance were notably enhanced. Feng et al. [26] tested cement-stabilized mixtures’ strength, frost resistance, water stability, and fatigue resistance with different molding methods and tailing sand contents. The findings revealed that the unconfined compressive strength, water stability, and frost resistance of cement-stabilized aggregates reached their peak when the content of iron ore tailings sand (IOTS) was 10%. Deng et al. [27] examined the influence of IOTS content on the performance of cement-stabilized crushed stone base pavements. They concluded that cement-stabilized crushed stone mixtures incorporating 10–20% IOTS could satisfy the performance criteria for road construction. Public fillers are mainly applied to road sub-base due to the high mortar component, high water absorption, and low strength on the surface. Due to the lack of variation in the patterns of mechanical properties, dry shrinkage properties, and freeze–thaw properties of public fill cement-stabilized aggregate (PF-CSA), the reasons affecting the properties must be analyzed from a microscopic point of view to improve the performance and application scope of recycled mixtures [28,29].

In order to precisely assess the impact of incorporating public fill (PF) on the properties of CSA and elucidate the underlying mechanisms governing the formation of mechanical properties, this study scrutinized variations in mechanical characteristics, shrinkage behavior, and durability of cement-stabilized recycled mixtures across diverse cement and incorporation rates. Moreover, this investigation aimed to delineate the mixing range and establish a design parameter index system tailored for low- and medium-grade road construction. Anticipated outcomes of this research encompass the facilitation of the effective deployment of PF-CSA in civil engineering projects, offering valuable insights into the development of eco-conscious road infrastructure and practical directives for the recycling and reclamation of construction waste.

## 2. Materials and Methods

### 2.1. Raw Materials

#### 2.1.1. Aggregate

**Figure 1:**
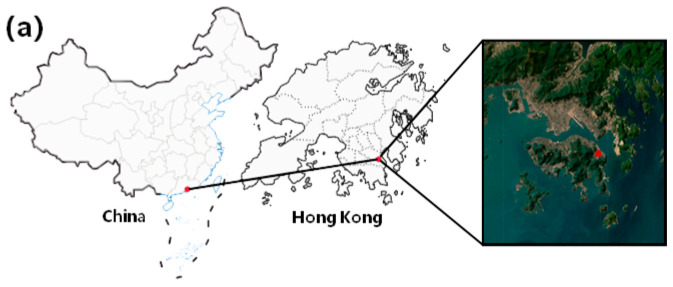

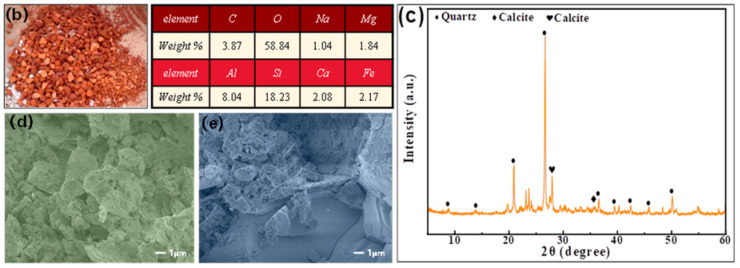
Overview of the project: (**a**) project location, (**b**) PF appearance, (**c**) XRD image of PF, (**d**,**e**) SEM images of PF.

The construction waste primarily originated from the demolition of old buildings during renovation projects in the offshore area of Hong Kong and mainly consisted of brick aggregates (Figure 1). The processing methodology involved a sequence of operations including crushing, manual sorting, impact crushing, and screening. Concrete waste and brick slag collected underwent drying and crushing to produce coarse and fine aggregates with varying particle sizes (4.75–9.5 mm, 9.5–19 mm, 19–31.5 mm), with basalt constituting the composition of the natural aggregate. Two types of aggregates, namely Natural Aggregate (NA) and Public Fill (PF), underwent testing based on raw material technical indicators, and the results are detailed in Table 1.

As depicted in Figure 1, the peak intensities of quartz and calcite in PF exhibited elevation, primarily attributed to the inclusion of natural aggregates within the parent concrete waste. Additionally, the incorporation of carbonated cement pastes within the parent concrete waste contributed to the heightened peak intensity of calcite (CaCO_3_); in addition, the low content of silicate can be further captured. The XRD images also revealed that PF contained a high porosity at all crystallographic levels with a high SiO_2_ peak strength. The SEM images revealed that PF has an irregular particle shape and higher porosity than natural aggregate due to the long-term process of weathering, erosion, and crushing. In addition, the surface of PF was covered with a significant amount of cement mortar and microcracks, resulting in a rougher microstructure that increased its water-absorption capacity and porosity while reducing its overall strength and durability.

#### 2.1.2. Cement

In this study, the delayed-setting cement (P.O 42.5) utilized was sourced from ZhongLian Cement Co., Ltd., Lianyungang, China. The principal parameters of the cement are delineated in Table 2, with all outcomes satisfying the stipulations outlined in the JTG/T F20-2015 specification [30].

### 2.2. Testing Methods

**Figure 2:**
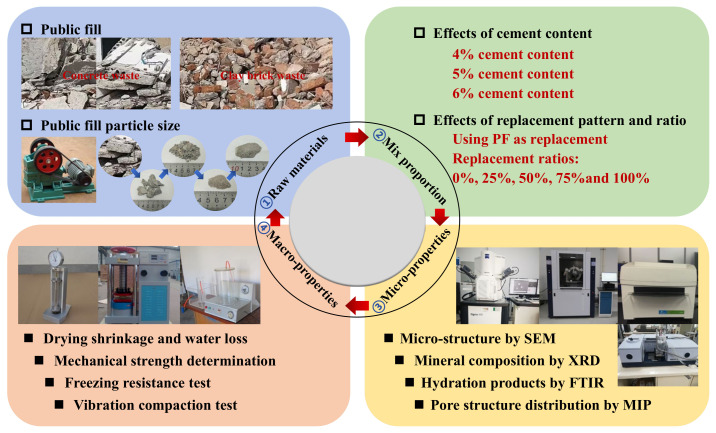
Flowchart of experiment.

The mix proportion design, specimen compaction test, specimen preparation and maintenance, unconfined compressive strength, splitting strength, compressive modulus, freeze-thaw resistance, and dry shrinkage tests were conducted in accordance with established specifications. The testing procedures and key apparatuses employed are illustrated in Figure 2.

#### 2.2.1. Mixture Design

**Figure 3:**
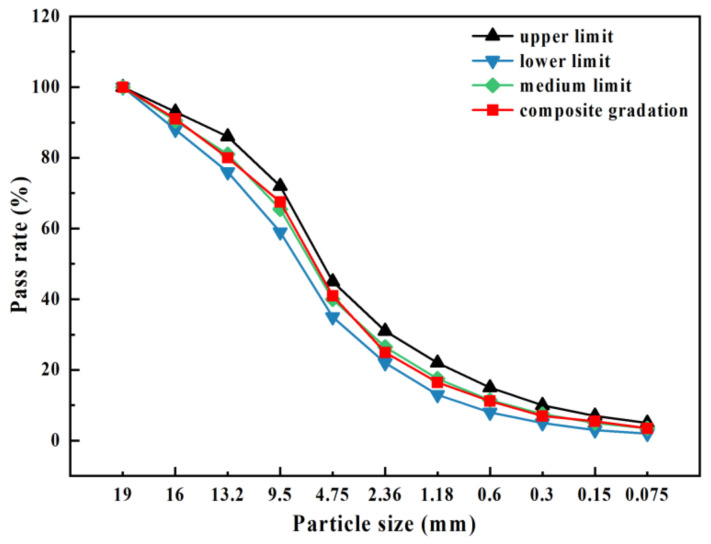
Grading design curve.

The cement-stabilized mixtures derived from construction waste must initially satisfy strength criteria. Previous research has demonstrated that cement-stabilized crushed stone with a compact skeleton exhibits superior compressive strength and modulus of elasticity compared to mixtures characterized by compact suspension and void skeletons. The grading design curve is shown in Figure 3. In order to adhere to the technical specifications outlined in the Technical Guidelines for Construction of Highway Road-bases (JTG/T F20-2015) for second-class highways [30], this study employed the median C-B-2 grading value indicative of the compact skeleton structure. Given the distinct densities of crushed basalt and public fill, volume was selected as an alternative parameter in this investigation to ensure grading uniformity.

#### 2.2.2. Test Design

The recycled fine aggregates from crushed concrete waste and red bricks have low strength and too high an organic content to be used in the test. Therefore, coarse aggregates (9.5–19 mm) were considered replacements, and natural crushed stones were adopted as fine aggregates in this test. Recycled cement-stabilized mixtures were mainly applied for compaction tests, strength, stiffness, drying shrinkage, and frost resistance tests with different PF contents (0%, 25%, 50%, 75%, and 100%) and different cement contents (4%, 5%, and 6%). The experiments were conducted in accordance with the Test Methods of Materials Stabilized with Inorganic Binders for Highway Engineering (JTG E51-2009) [31], with detailed test parameters outlined in Table 3.

#### 2.2.3. Sample Preparation and Curing

Cylindrical specimens measuring 150 mm in diameter and 150 mm in height were subjected to tests for unconfined compressive strength, splitting strength, compressive rebound modulus, and freeze–thaw cycles. The compaction control index was set at 98%. Dry shrinkage tests were conducted on beam specimens measuring 100 mm × 100 mm × 400 mm. Specimens with curing periods less than 28 days underwent standard curing conditions, while those with curing periods exceeding 28 days were subjected to 90 days of curing in a dry shrinkage chamber (maintained at 20 °C ± 2 °C, with 60% humidity) following 28 days of standard curing.

#### 2.2.4. Mechanical Property Test

The test specimens underwent a standard curing duration of 7 days. On the final day of the curing period, the specimens were submerged in water, with the water level approximately 2.5 cm above the upper surface of the specimens to ensure effective immersion. The unconfined compressive strength and splitting strength were determined using an electro-hydraulic servo universal testing machine (WAW-1000, Shanghai Bairoe Test Instrument Co., Ltd., Shanghai, China), with a loading speed set at 1 mm/min. Mean values and coefficients of variation were computed to ascertain representative values.

#### 2.2.5. Compressive Rebound Modulus

The elastic modulus of compression was assessed in accordance with the T0808-1994 standard (top surface method), with the specimens aged for 90 days. To mitigate the influence of testing machine vibrations on micrometer data collection, an electro-hydraulic servo universal testing machine (WAW-1000) was employed.

#### 2.2.6. Freeze–Thaw Test

The freeze–thaw experiments adhered to the specifications outlined in T0858-2009. Specimens were aged for 28 days. Following five freeze–thaw cycles, the unconfined compressive strength RDC of the specimens was assessed and compared with that of the unfrozen specimens. Subsequently, the frost resistance index BDR was calculated.

#### 2.2.7. Dry Shrinkage Test

In accordance with the T0854-2009 standard, the beam specimens underwent a standard curing process within a curing box for 6 days, followed by immersion in water for 1 day. Subsequently, the specimens were transferred to a drying shrinkage chamber for conducting the drying shrinkage test using a multimeter.

#### 2.2.8. Micro-Properties Characterization

A series of micro experiments were conducted to elucidate the microscopic characteristics of construction waste as potential aggregate substitutes, aiding in the understanding of its macro-performance. Specimens measuring 50 mm × 50 mm were sampled and treated with isopropanol to halt hydration. Scanning electron microscopy (SEM) was employed to examine the microscopic morphology and microstructure of recycled cement-stabilized macadam with varying proportions of PF. X-ray diffraction (XRD) analysis within the range of 5–60° (2θ) was utilized to identify the mineral composition and crystal phases of PF and its recycled cement-stabilized aggregates. Fourier transform infrared spectroscopy (FTIR) with a wavenumber range of 400–1600 cm^−1^ was employed to analyze the chemical bonding vibration and reaction mechanism of the recycled cement-stabilized aggregates incorporating public filler. Furthermore, the pore structure of the paste was assessed using Mercury Intrusion Porosimetry (MIP) with an automated mercury porosimeter (Autopore IV 9510, Monitor Instrument Co., Ltd., Shenzhen, China).

## 3. Results and Discussion

### 3.1. Vibration Compaction Test

**Figure 4:**
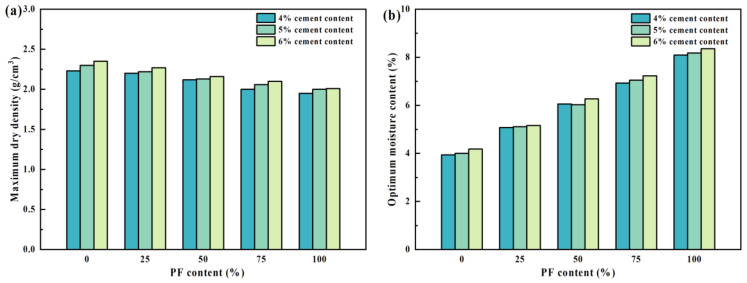
The results of the vibration compaction test. (**a**) Maximum dry density; (**b**) Optimum moisture content.

The findings from vibratory compaction are depicted in Figure 4. The optimal moisture content of the mixtures exhibited an upward trend with increasing PF content under the same cement dosage. Specifically, a 25% increase in PF content resulted in a rise of approximately 1% to 1.5% in the optimal moisture content, coupled with a decrease in the maximum dry density of the mixture by approximately 0.05 g/cm^3^ to 0.12 g/cm^3^. These alterations can be primarily attributed to the aggregate density dictating the maximum dry density of the mixtures. The cement mortar enveloping the PF surface possesses high porosity and a lower density compared to natural gravel. Consequently, the water absorption capacity of the cement mortar surpasses that of gravel, leading to internal damage within the recycled aggregate during crushing, thereby causing a decline in maximum dry density as the optimal moisture content increases with higher PF content.

### 3.2. Unconfined Compressive Strength

**Figure 5:**
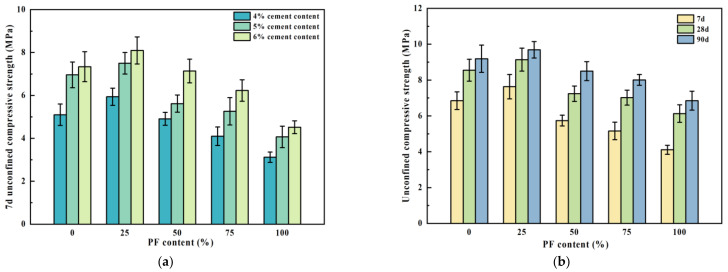
Unconfined compressive strength of the public fill mixture. (**a**) 7d unconfined compressive strength; (**b**) unconfined compressive strength.

The 7-day, 28-day, and 90-day unconfined compressive strengths of recycled cement-stabilized aggregates with varying PF contents are illustrated in Figure 5. Across cement dosages of 4%, 5%, and 6%, the 7-day unconfined compressive strength of the mixtures exhibited a gradual rise followed by a subsequent decline with increasing PF content. The 7-day unconfined compressive strength reached its peak at 25% PF content. Specifically, at cement dosages of 4%, 5%, and 6%, the 7-day unconfined compressive strength increased by 14.1%, 7.2%, and 9.4% respectively, when the PF content was 25% compared to 0%. This trend arises from the rough surface of PF, which allows for attachment of anhydrous cement mortar. PF also contains reactive substances such as silica and calcium oxide, which promote pozzolanic reactions and enhance interfacial bonding. However, when the PF content exceeded 25%, the strength of the composite mixture declined notably due to the increased binding force of PF, which compromised the overall strength. According to the Technical Guidelines for Construction of Highway Road-bases (JTG/T F20-2015) [30], the 7-day unconfined compressive strength of cement-stabilized materials for highways and primary roads should fall within the range of 4.0~6.0 MPa under heavy traffic conditions. A 7-day unconfined compressive strength of 5% can satisfy these specifications. Consequently, performance tests for other cement-stabilized regenerated mixtures were conducted with a 5% cement content.

The unconfined compressive strengths of the recycled mixtures, with a 5% cement content, exhibited an initial increase with the augmentation of PF content, followed by a gradual decline, peaking at 25% PF content. In comparison with mixtures devoid of PF (0% content), those containing 25% PF showed respective increases in compressive strengths at 7 days, 28 days, and 90 days by 10.2%, 6.5%, and 5.1%, respectively. Conversely, as the PF content rose to 100%, the unconfined compressive strengths at 7 days, 28 days, and 90 days decreased by 46.1%, 32.9%, and 29.3% respectively. It is hypothesized that when the PF content exceeds 25%, the skeleton structure of the mixture changes from skeleton-dense to suspension-dense, drastically reducing the compressive strength.

### 3.3. Splitting Strength

**Figure 6:**
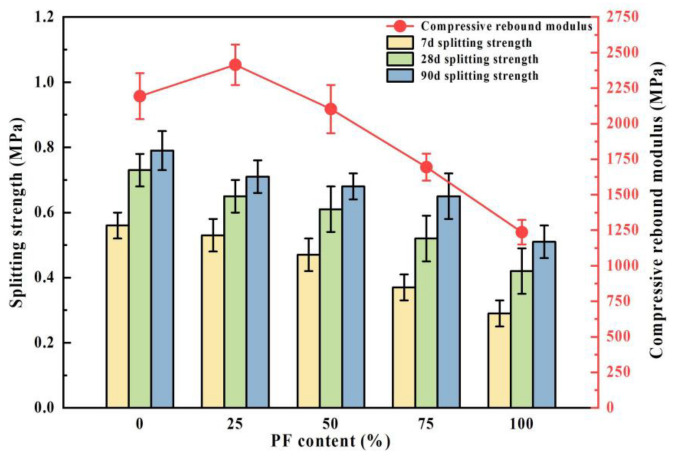
Splitting strength test and compressive rebound modulus of the public fill mixture.

The outcomes of the splitting strength test conducted on cement-stabilized recycled mixtures incorporating PF are depicted in Figure 6. Across the 7-day, 28-day, and 90-day intervals, the splitting strengths of the mixtures exhibited a notable decrease as the PF content decreased. Specifically, compared to mixtures devoid of PF (0% content), the 7-day, 28-day, and 90-day splitting strengths decreased by 28.6%, 17.8%, and 19.5%, respectively. This decline can be attributed to the larger crushing value of PF relative to NA, leading to compression-induced crushing of aggregates during standard specimen formation. Consequently, a shift in particle size occurred, resulting in fewer coarse aggregates and an increase in finer aggregates. Additionally, a small amount of cement paste adhering to the aggregate crushing interface represents a weak surface within the mixture under stress, prone to slipping along the failure interface under splitting force. Consequently, higher PF content contributes to weaker interfacial regions and diminished splitting strength [32].

At a 5% cement dosage, the splitting strength of the recycled mixtures exhibited a significant increase from 7 days to 28 days, followed by a slower increase from 28 days to 90 days, consistent with the compressive properties of cement-stabilized materials. Relative to the 7-day splitting strength, the average 28-day splitting strength of the mixtures increased by 24.2%, while the 90-day compressive strength increased by 33.5%. The 28-day splitting strength of the mixtures with varying PF dosages accounted for 92.4%, 91.5%, 89.7%, 80.2%, and 82.3% of the 90-day strength, respectively, suggesting that the splitting strength of the recycled mixtures primarily develops within the initial 28 days of the curing period. The pattern of splitting strength change with age mirrored that of compressive strength, suggesting a shared underlying mechanism.

### 3.4. Compressive Rebound Modulus

**Figure 7:**
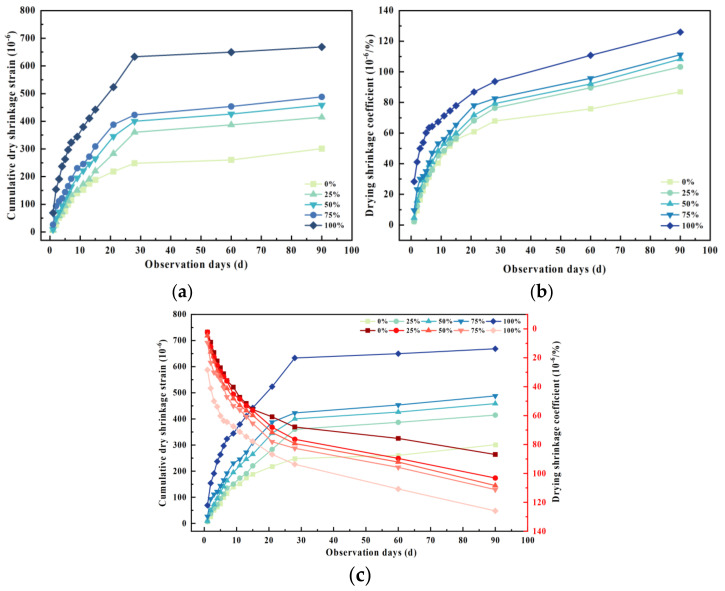
Drying shrinkage test of the recycled mixture. (**a**) Cumulative dry shrinkage strain; (**b**) dry shrinkage loss rate; (**c**) drying shrinkage strain.

The 90-day compressive rebound modulus of cement-stabilized recycled mixtures is depicted in Figure 7. Compressive elastic modulus serves as an indicator of a pavement material’s resistance to vertical deformation. With an increase in PF content, the compressive rebound modulus of the recycled mixture exhibited a pattern of initial augmentation followed by a decline, consistent with the trend observed in the unconfined compressive strength of the recycled mixture, peaking when the PF content reached 25%. This phenomenon can be attributed to the considerable presence of cement mortar on the surface of PF, characterized by substantial porosity. Moreover, the mechanical crushing process induces an increase in microcracks and porosity within the mixture, consequently leading to a reduction in the compressive elastic modulus with increasing PF content [33]. Nevertheless, the incorporation of some PF resulted in a rougher mixture surface compared to crushed gravel, thereby enhancing the internal frictional resistance of the mixture and promoting densification and integrity. Consequently, the compressive elastic modulus of the mixture reached its maximum value at a PF content of 25%.

### 3.5. Dry Shrinkage Property

The dry shrinkage strain and dry shrinkage coefficient of cement-stabilized mixtures with varying PF contents, alongside 5% cement content, are depicted in Figure 7. The dry shrinkage process can be delineated into three stages: (i) a rapid phase within the initial 7 days; (ii) a gradual phase spanning from 7 to 30 days; and (iii) a stabilized phase post-30 days. At the 90-day mark, the dry shrinkage strain of specimens increased by 117.5%, while the dry shrinkage coefficient surged by 31.95% in comparison with specimens containing 100% and 0% PF content. This trend indicates that augmenting PF content may exacerbate the dry shrinkage performance of the mixture, necessitating thorough consideration in pavement structure design. This is primarily attributable to PF’s high porosity, elevated water absorption, low density, and increased mortar adhesion on its surface. PF aggregates generate numerous microcracks during crushing and processing, thereby escalating the water content of the recycled mixture, while the heightened porosity affords greater space for shrinkage and deformation.

Under a fixed cement dosage, the dry shrinkage strain and dry shrinkage coefficient of mixtures with varying PF content exhibit a distinct variation pattern over test duration, with both parameters gradually increasing with test days. During the initial 7 days, dry shrinkage strain and dry shrinkage coefficient escalate rapidly, with the former reaching approximately 50% and the latter about 60% of the final coefficient. From the 7th to the 30th day, the rate of increase in dry shrinkage strain and dry shrinkage coefficient continues to diminish, registering a slower pace than the initial stage. These findings underscore the dominance of dry shrinkage changes in recycled mixtures with differing PF contents, emphasizing the necessity of meticulous water application during initial pavement base construction. The dry shrinkage properties of recycled mixtures containing 25% PF content closely resemble those of conventional cement-stabilized mixtures devoid of PF, underscoring improved drying shrinkage properties when PF content remains below 20%.

### 3.6. Freezing Resistance Test

**Figure 8:**
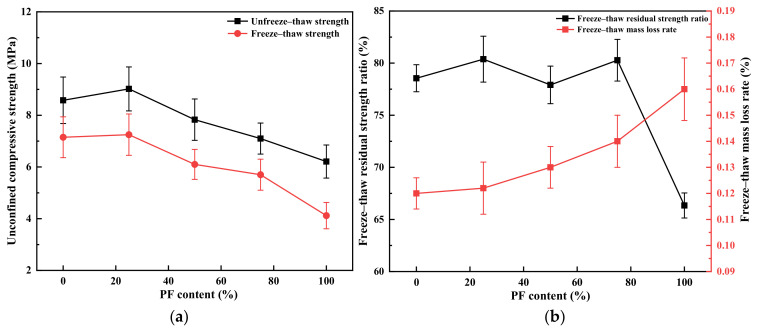
Frost resistance test of the PF mixture. (**a**) Unconfined compressive strength; (**b**) residual ratio of freeze–thaw strength.

As the content of PF increases, the mixtures exhibit notable water absorption, leading to increased expansion pressure during low-temperature conditions. Hence, an examination of the frost resistance of mixtures with varying levels of public filler content becomes imperative. The test outcomes of cement-stabilized recycled mixtures featuring different PF contents, subjected to five freeze–thaw cycles, are presented in Figure 8. The residual strength ratio post-freezing and thawing gradually diminishes with rising PF content, accompanied by a progressive increase in the rate of mass loss due to freezing and thawing. Relative to mixtures devoid of PF content, the residual strength ratios after five freeze–thaw cycles decline by 2.92%, 5.39%, 7.24%, and 16.91% for recycled mixtures containing PF contents of 25%, 50%, 75%, and 100%, respectively. This trend is primarily attributed to the incorporation of substantial PF quantities, coupled with the attachment of cement mortar to PF surfaces characterized by significant porosity. Consequently, higher PF content results in increased water absorption during immersion, subsequently amplifying expansion pressure during freezing. The heightened expansion pressure, along with an augmented weak interfacial transition zone, leads to extrusion damage within the specimen’s pores and interfacial transition zone [34]. The multiple cycles of freeze–thaw action exacerbate internal specimen damage, ultimately reducing compressive strength. The impact escalates proportionately with the increase in PF content. Based on the frost resistance requirements outlined in the Technical Guide for Frost Resistance Design and Construction of Highway Engineering, the mixtures can all satisfy the frost protection performance mandates for semi-rigid base pavement mixtures.

### 3.7. Mechanism Analysis

#### 3.7.1. XRD Analysis

**Figure 9:**
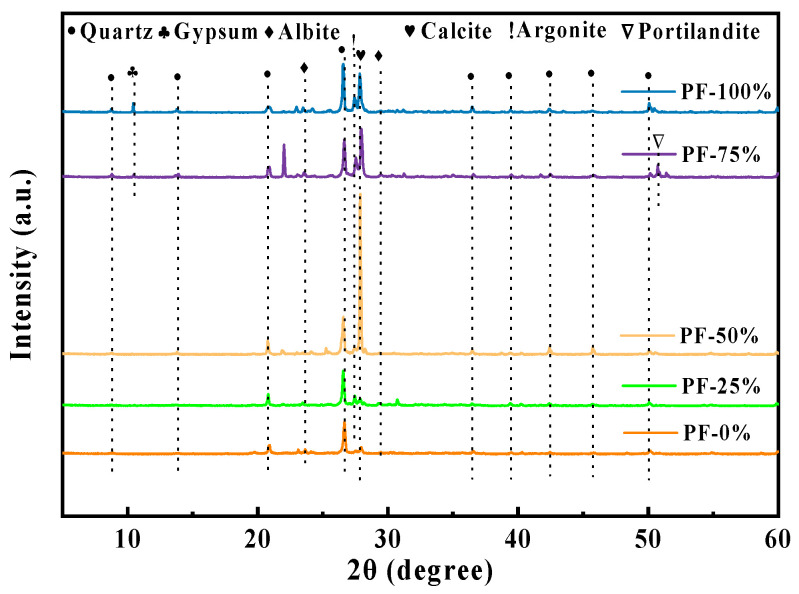
XRD pattern of PF mixture.

Figure 9 illustrates the XRD pattern depicting the substitution of basalt PF. The partial replacement of NA with PF augmented the peak intensity of SiO_2_, while the inclusion of PF bolstered the strength of quartz and calcite within the CSA. Conversely, the intensity of mullite decreased due to the prevalence of quartz and calcite in PF. Comparison of the XRD patterns between CSA formulations prepared with 25% and 75% PF revealed a reduction in cementitious products in CSA mixed with 75% PF, attributed to the inert nature of quartz in PF, which minimally participates in the reaction, thereby reducing the generation of cementitious products in the alkali-activated paste. In addition, gypsum was formed when the PF dosage reached 75% and 100%. Ca(OH)_2_ and gypsum could interact in a secondary hydration reaction with the active ingredients in the mixture, such as activated silica and activated alumina [35,36].

Upon substituting PF for a portion of NA, the addition of PF resulted in a reduction in active components within the blended binder while concurrently promoting the formation of secondary hydration products. This led to a noticeable decrease in the intensity of Ca(OH)_2_ in the paste, aligning with findings reported by Luo et al. [37] and Wu et al. [38]. Rovnaník et al. [39] indicated that the amorphous phase content in PF was approximately 27.8 wt% and 21.75 wt%, respectively, attributed to heat treatment causing dehydroxylation of clay minerals and the formation of reactive amorphous phases in clay brick. The substitution of NA with PF facilitated the participation of amorphous SiO_2_ and Al_2_O_3_ in a hydrocolloidal reaction, resulting in the consumption of existing Ca(OH)_2_. Therefore, the addition of PF as an aggregate replacer diminished the intensity of Ca(OH)_2_ in the mortar paste.

#### 3.7.2. SEM Analysis

**Figure 10:**
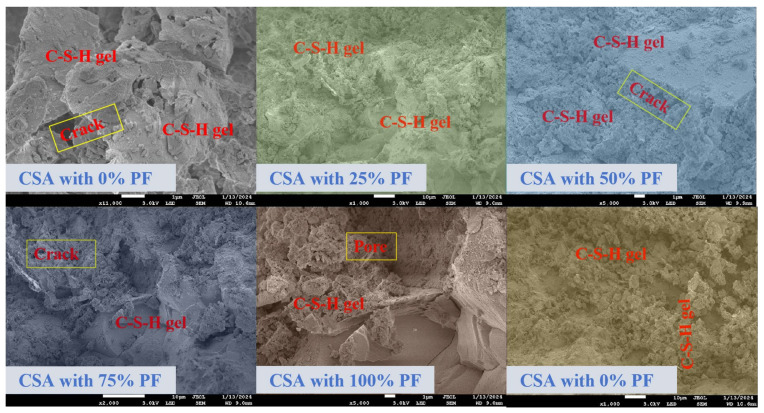
SEM observation of PF mixture.

Figure 10 presents SEM observations of CSA with varying PF contents. The unmodified slurry devoid of PF exhibited a compact microstructure characterized by prominent C-S-H gels. With increasing substitution rates of PF from 0% to 100%, the PF particles became less encapsulated with C-S-H gel, resulting in a more extensive network of pores within the microstructure of the recycled aggregates. The incorporation of these aggregates into CSA weakened the bond between the existing interfacial transition zone (ITZ) and the new ITZ, consequently compromising the mechanical properties of the concrete. Furthermore, during the hydration reaction, fresh hydration products emerged and diffused within the surface region of the PF particles. Cement pastes containing 25% PF displayed robust and effective binding between PF particles and the newly formed hydration products. The paste containing unhydrated cement clinker remained unaffected by the replacement of small amounts of NA with PF, preserving its microstructure. However, the complete replacement of NA with 100% PF, i.e., ultra-high substitution of PF in cement, could hinder microstructure development and weaken the bond between PF particles and the C-S-H gel. 

#### 3.7.3. FTIR and MIP Analysis

**Figure 11:**
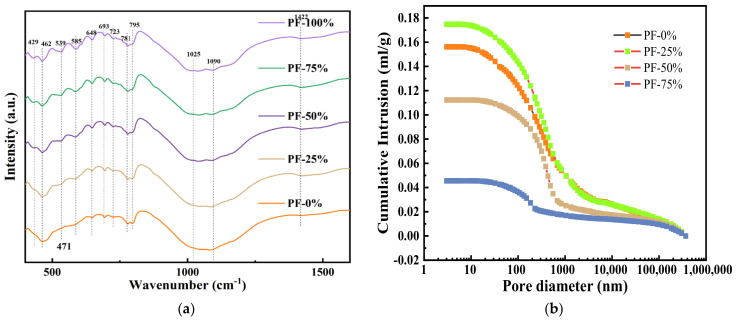
FTIR spectrum (**a**) and MIP results (**b**) of PF mixture.

The FTIR findings of CSA with varying PF contents are depicted in Figure 11a. The wave number associated with Si-O bending vibration, around 460~465 cm^−1^, exhibited an increase with the inclusion of PF, while the blending of PF reduced the wave number related to Si-O stretching vibration, approximately near 781 cm^−1^, indicating a reduction in generated cementitious products in PF-CSA. The high concentration of CaCO_3_ in PF led to increased wave number and absorption peak of C-O vibration, observed near 874 cm^−1^ and 1422 cm^−1^, respectively, with the addition of PF as an aggregate replacement. Furthermore, substituting partial NA with PF increased the wave number of Si-O-Si stretching vibrations near 973 cm^−1^, signifying enhanced hydration product formation, in line with XRD analysis.

The pore size distribution of CSA mixed with PF is illustrated in Figure 11b. The incorporation of PF augmented the average pore size of CSA; specifically, the average pore size of CSA mixed with 0% PF and 25% PF increased by 9.7% and 27.2%, respectively. Moreover, replacing 100% PF with NA led to a substantial increase in the pore size of the alkali-activated slurry. The average pore size of CSA including 100% PF was 3.5 times larger than that of CSA alone. PF comprises a significant proportion of inert components, contributing to the loosening of the microstructure of the prepared CSA. While inert quartz and calcite constitute the majority of CSA with 100% PF, these components exhibit insensitivity to reaction in CSA, resulting in a notable increase in the pore size of CSA. Huseien et al. [40] and Ulugöl et al. [41] have previously elucidated that quartz and calcite, being structurally stable, pose challenges in dissolution processes.

## 4. Conclusions

This study investigated the road performance of the recycled mixture by examining the unconfined compressive strength, splitting strength, compressive resilience modulus, dry shrinkage, and frost resistance. Additionally, the impact of incorporating PF at various types and replacement ratios on the microstructure of CSA was explored. This approach provides a practical solution for recycling increasing amounts of construction waste and aligns with “Low Carbon and Green” standards for transport infrastructure construction. The conclusions are summarized as follows:

1. The unconfined compressive strength of cement-stabilized recycled mixture with varying PF content met the base strength requirements of heavy, medium, and light traffic pavement on secondary and sub-secondary roads in China. The unconfined compressive strength and resilience modulus followed a similar trend, peaking at 25% PF content, indicating excellent filler material properties of public fill.
2. With an appropriate PF content, paste reaction products mainly comprised C(N)-A-S-H, hydrotalcite, Ca(OH)_2_, and CaCO_3_. As PF replacement increased from 0% to 25%, gel products gradually stacked and cemented together with unreacted particles, improving microstructure compactness. Formation of these components reduced dry shrinkage strain and effectively inhibited reflection crack formation. However, when PF content increased from 50% to 100%, unreacted PF increased and adhered to the surface of the reaction product, resulting in a loose and rough microstructure.
3. Creating an affordable, value-added, multi-component material in civil engineering that extensively incorporates construction waste can effectively mitigate waste accumulation issues. Furthermore, it introduces innovative civil construction materials that yield substantial social, economic, and environmental advantages.

## Figures and Tables

**Table 1:** Raw material technical index of coarse aggregate.

Detection Index	NA		PF		Standard Value
	5–10	10–20	5–10	10–20	
Crushing value (%)	19.5	15.6	28.4	25.9	30
Apparent density (kg/m^3^)	2.91	2.88	2.54	2.35	—
Water absorption (%)	2.35	1.37	11.95	6.76	—
Needle flake content (%)	6.52	5.49	5.12	4.75	≤20
Mud content (%)	0.67	0.51	1.93	1.15	≤2.0

**Table 2:** Cement performance.

Detection Index	Chemical Composition (%)			Setting Time (min)		Linear Expansion Rate (%)		Compressive Strength (MPa)		Flexural Strength (MPa)	
	MgO	SO_3_	CaO	Initial Setting Time	Final Setting Time	3d	28d	3d	28d	3d	28d
Detection value	2.32	2.91	0.87	311	412	0.15	0.32	27.1	46.3	5.3	7.8
Standard value	≤5.0	≤3.5	≤1.0	≥300	≤720	≥0.1	≤0.5	≥17.0	≥42.5	≥3.5	≥6.5

**Table 3:** Mix proportions of recycled cement-stabilized macadam.

Sample	Cement (%)	Basalt Aggregate (%)	Public Fill (%)
M-Control-4	4	100	0
M-Control-5	5		
M-Control-6	6		
M-PF-25	4	75	25
	5		
	6		
M-PF-50	4	50	50
	5		
	6		
M-PF-75	4	25	75
	5		
	6		
M-PF-100	4	0	100
	5		
	6		

## Data Availability

The raw data supporting the conclusions of this article will be made available by the authors on request.
